# Carbon nanomaterials

**DOI:** 10.3762/bjoc.10.186

**Published:** 2014-08-05

**Authors:** Anke Krueger

**Affiliations:** 1 Institute for Organic Chemistry, Julius Maximilians University Würzburg, Am Hubland, 97074 Würzburg, Germany; 2 Wilhelm Conrad Röntgen Research Center for Complex Material Systems (RCCM), Julius Maximilians University Würzburg, Sanderring 2, 97070 Würzburg, Germany

**Keywords:** carbon allotropes, carbon nanomaterials, carbon-rich molecules

The era of carbon nanomaterials has started with the first reports on fullerenes and related compounds in the mid-eighties, and a tremendous increase of the research activity in the field has been observed ever since. New classes of carbon materials have entered the scene such as carbon nanotubes, carbon onions, nanoscale diamond and diamondoids. A major turning point was the appearance of graphene as a material available for in-depth investigations – spurred by the development of reliable production methods. The progress in terms of understanding the properties and chemistry of carbon nanomaterials has opened a whole new world of applications for nanomaterials in general.

The reliable production of a material represents a necessary requirement for the development of a research field. By now, all types of carbon nanomaterials and many carbon-rich organic materials are available to the scientific community in excellent quality and suitable amounts for the investigation of fundamental properties and prospective applications. This has led to the emergence of a new community of scientists working in an interdisciplinary area involving materials science, organic chemistry and physics.

Synthetic organic chemistry is a major part of carbon materials chemistry as the rational synthesis of carbon allotropes such as fullerenes and nanotubes and related molecular compounds with and without heteroatoms remains a challenging task. The contribution does not end here but continues with the development of suitable reactions for the controlled functionalization of molecular as well as nanoscale allotropes such as fullerenes, diamondoids, carbon onions, carbynes or nanodiamonds. The interplay with knowledge gained by materials scientists and physicists on the electronic or optic properties of materials enables the fine-tuning of materials properties and the conjugation of functional moieties with the carbon nanomaterial. This interdisciplinary approach opened the door to a broad range of applications including energy conversion and storage, catalysis, electronic and optoelectronic as well as biomedical applications.

The present Thematic Series attempts to showcase the diversity in the field of carbon nanomaterials and carbon-rich compounds and to emphasize the links between the different classes of materials. Contributions from synthetic organic chemistry dealing with the formation and functionalization of carbon-rich molecules and from the area of functional nanomaterials illustrate the plethora of research activities. Obviously, such an issue will fail to present the field in its entirety. The sheer number of publications appearing globally every year attests to this. However, a topical collection enables a focused view on some of the novel developments.

I would like to thank all authors for their excellent contributions to this Thematic Series. The publication of the manuscripts would not have been possible without the contribution of detailed and timely reports by reviewers from all fields of carbon chemistry. Furthermore, I would like to thank the editorial team at the Beilstein-Institut for their support and hard work. The open access policy of the Beilstein Journal of Organic Chemistry facilitates the availability of the articles of this Thematic Series to researchers and interested people all over the world, thus enabling a broad discussion of the topics.

Anke Krueger

Würzburg, June 2014

